# Postpartum-onset anti-PM/Scl–positive dermatomyositis–systemic sclerosis overlap syndrome with reversible interstitial lung disease: a case report

**DOI:** 10.3389/fmed.2026.1848783

**Published:** 2026-07-01

**Authors:** Joud Zghyer, Asad Omarion, Leen Zghyer, Yaman Ayasa, Omar Al-Deek, Adnan A. M. Wahdan

**Affiliations:** 1 Faculty of Medicine, Al-Quds University, Jerusalem, Palestine; 2 Department of Internal Medicine, Palestine Medical Complex, Ramallah, Palestine

**Keywords:** dermatomyositis, interstitial lung disease, overlap syndrome, postpartum, rituximab, systemic sclerosis

## Abstract

**Background:**

Polymyositis–scleroderma autoantibody (Anti-PM/Scl) associated connective tissue disease is a recognized overlap syndrome classically characterized by features of polymyositis- systemic sclerosis overlap. However, presentation with a dermatomyositis (DM) phenotype accompanied with interstitial lung disease (ILD) is less commonly reported. Pregnancy and the postpartum period are recognized immunological triggers for overlap syndromes, potentially leading to autoimmune disease. Postpartum-onset inflammatory myopathy with overlap features and significant pulmonary involvement poses substantial diagnostic challenges due to its heterogeneous clinical presentation and evolving serologic profiles.

**Case presentation:**

We present a case of a 25-year-old Palestinian woman presenting with progressive proximal muscle weakness, characteristic dermatomyositis cutaneous manifestations, notable unintentional weight loss, and systemic symptoms 4 months postpartum. Laboratory tests showed markedly elevated creatine kinase and positive antinuclear antibodies, positive anti-PM/Scl antibodies and positive anti-dsDNA antibodies, while anti-U1-RNP antibodies were negative. Imaging demonstrated hepatomegaly and splenomegaly, and pulmonary function testing showed interstitial lung disease (ILD). Muscle biopsy confirmed inflammatory myopathy without typical perifascicular atrophy. The patient showed incomplete clinical response to treatment with corticosteroids and mycophenolate mofetil; however, transitioning to rituximab resulted in substantial clinical, pulmonary, and functional improvement.

**Conclusion:**

This case illustrates a diagnostically challenging presentation of postpartum-onset Anti-PM/Scl–Positive dermatomyositis–systemic sclerosis overlap syndrome complicated by ILD and multisystem involvement. It underscores the diagnostic challenges associated with overlap syndrome and connective tissue disease phenotypes, and supports the potential effectiveness of rituximab in refractory overlap myositis and reversing inflammatory ILD.

## 1 Introduction

Connective tissue diseases (CTD) are systemic autoimmune diseases characterized by heterogeneous clinical features and multisystem involvement. These Disorders are classified according to internationally recognized diagnostic criteria and are often associated with specific serologic markers to distinguish them, some of the CTDs include rheumatoid arthritis (RA), systemic lupus erythematosus, polymyositis (PM) and systemic sclerosis (SSc), and many others (1). Despite the widely established classification criteria for CTDs, some patients exhibit clinical features that fulfill the diagnostic criteria of two or more distinct autoimmune disorders, a phenomenon known as overlap syndromes which is frequently encountered in clinical practice (2). Because manifestations of overlap syndromes span multiple organ systems and often evolve rather than present simultaneously, diagnosis can be challenging and is often delayed (3). Anti-PM/Scl antibodies are considered specific markers for systemic sclerosis and polymyositis overlap syndrome. Alongside myositis, positive anti-PM/Scl is reported to be associated with multiple clinical manifestations like Raynaud’s phenomenon, ILD, arthritis and cutaneous involvement (mechanic’s hand) (4). These overlap phenotypes exhibit heterogeneous clinical and serologic manifestations, often contributing to diagnostic complexity (5). Pregnancy and the postpartum period are recognized as high risk windows for the development and unmasking of autoimmune diseases, likely driven by profound hormonal and immunologic shifts as well as fetal microchimerism which has been particularly related to systemic sclerosis (6). Postpartum-onset inflammatory myopathies presenting as overlap syndromes with pulmonary and multisystem involvement are infrequently described in the literature, and their recognition poses significant diagnostic challenges.

Here, we report the case of a 25-year-old woman. This case is clinically instructive in its coexistence of positive anti-PM/Scl antibodies, DM phenotype, multisystem involvement, and inflammatory ILD within a single overlap syndrome presentation, particularly in the postpartum setting. To contextualize this presentation, we also review previously reported related cases and studies of overlap syndromes and dermatomyositis.

## 2 Case presentation

### 2.1 Patient information

The patient is a 25-year-old female, who was seen in our rheumatology clinic in Mid-2025 with no previous history of chronic rheumatological diseases. Her significant medical history includes the second pregnancy, during which the patient had episodes of respiratory distress, necessitating hospitalization following an Influenza B infection that had been confirmed by polymerase chain reaction (PCR).

### 2.2 Presenting complaints

She was admitted with symptoms of progressive proximal muscle weakness, difficulty rising from a seated position, climbing stairs, generalized myalgia, arthralgia, and skin changes. She had unintentional weight loss of approximately 22 kg within 6–8 months. She also mentioned various complaints, such as fatigue, dysphagia, lack of appetite, and intolerance to exercise.

### 2.3 History of present illness

Symptoms began approximately 4 months after her second delivery. She noted mild muscle aches and fatigue, which gradually progressed to difficulty with routine activities, such as climbing stairs, rising from a chair, or walking for long distances. Over subsequent months, she developed dermatomyositis cutaneous features, including hyperkeratotic fissuring of the hands, a violaceous periorbital rash, and papular skin lesions on her hands, consistent with a heliotrope rash and Gottron’s papules. She also noted cold-induced color changes in her fingers (suggestive of Raynaud’s phenomenon) and progressive puffiness of her hands. Despite a trial of symptomatic therapy (high dose corticosteroids), her weakness worsened, and she began to lose weight rapidly, along with loss of appetite, dysphagia and increasing fatigue, prompting repeated hospital admissions.

### 2.4 Past medical, obstetric, and social history

During pregnancy, the patient experienced Influenza B infection complicated by respiratory distress requiring intensive care unit (ICU) admission. The patient also noticed diffuse scaly plaques during pregnancy that were initially diagnosed as psoriasis. These symptoms persisted alongside respiratory system involvement with dyspnea. These dermatologic manifestations were retrospectively reevaluated as potential indicators of early dermatomyositis.

She denied tobacco or illicit drug use, second-hand smoke exposure, toxin or occupational exposures, or prior chronic illness. No known family history of CTD was reported. There was no prior dermatologic, musculoskeletal, renal, or cardiovascular disease.

### 2.5 Physical examination (initial rheumatology evaluation)

**FIGURE 1:**
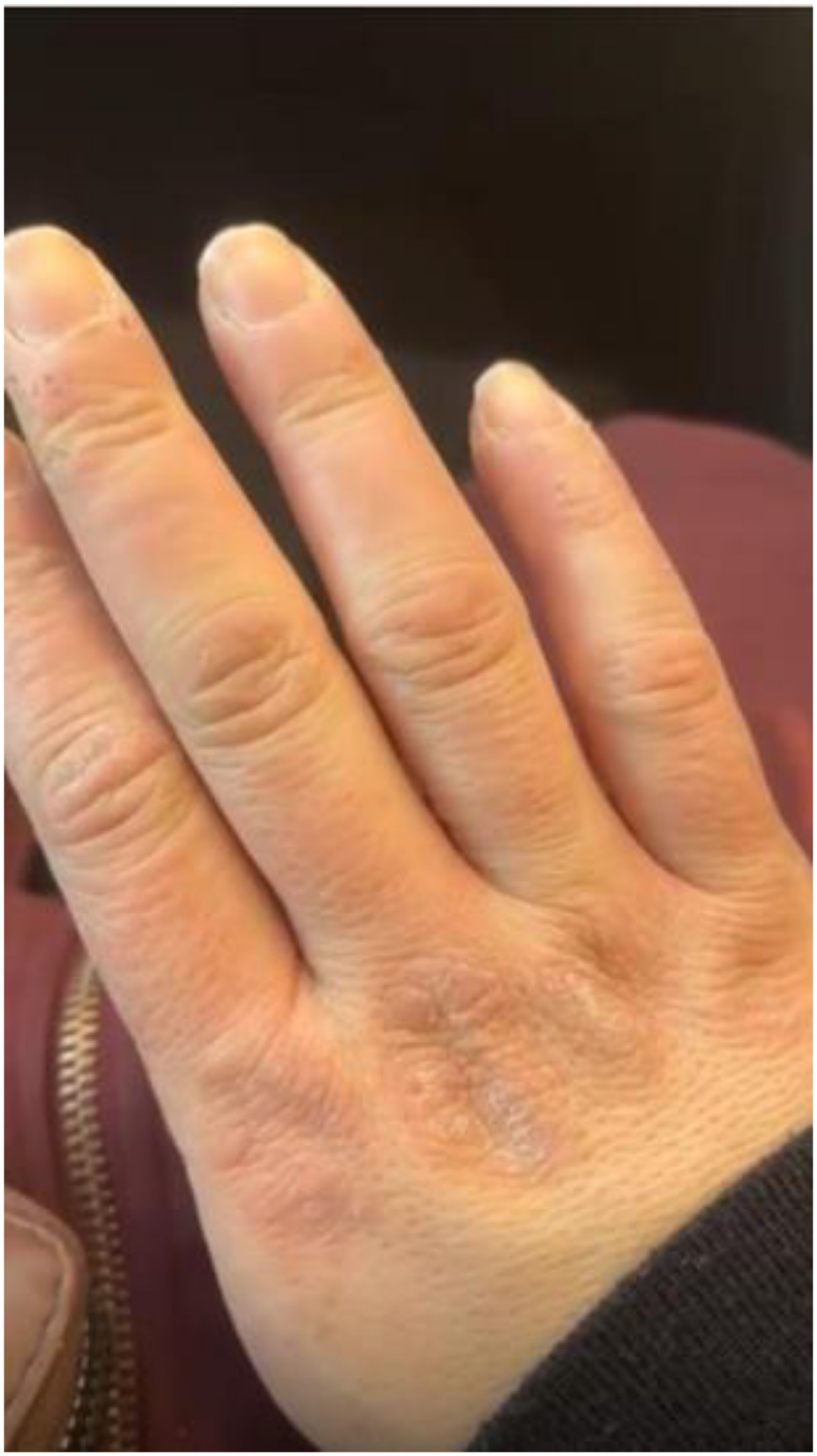
Gottron’s papules over the dorsal aspects of the metacarpophalangeal and interphalangeal joints.

On physical examination, the patient appeared cachectic. Hand examination showed cracked hyperkeratotic hands Gottron’s papules with visible nail fold telangiectasia and puffy hands without frank sclerodactyly (Figure 1). A malar rash and heliotrope rash were observed. Violaceous symmetric plaques were visible in the thighs and knees, and hyperkeratotic feet plantars.

She had symmetric proximal muscle weakness of upper and lower limbs on muscle strength examination, difficulty rising from the seated position, and a waddling gait. No clinical synovitis, or active arthritis were detected. No palpable lymphadenopathy, synovial thickening, digital ulcers, or skin tightening elsewhere.

At follow-up evaluation after treatment, the patient had marked improvement with 5/5 muscle strength in all extremities.

### 2.6 Investigations

#### 2.6.1 Laboratory findings

Laboratory data obtained from the patient’s hospital records revealed markedly elevated muscle enzymes and evidence of systemic inflammation. The total creatine kinase level initially was 4,000 U/L level (Reference Range 0–170 U/L) consistent with severe muscle injury, autoimmune tests revealed positive ANA antibodies at 1:320 (titer) and elevated rheumatoid factor mildly elevated at 48 IU/mL. Inflammatory markers showed marked elevation as well, the erythrocyte sedimentation rate (ESR) level was 22.0 Mm/h (Reference Range 0–10) Mm/h and C-reactive protein (CRP) levels were 21.4 mg/L (Reference Range 0–5) mg/L.

#### 2.6.2 Autoimmune serology

Subsequent testing revealed persistent rise in ESR and CRP levels, mild elevation in liver enzymes, and low Vitamin B_12_ levels. Complement levels were within the normal range. Autoimmune serology was positive for Antinuclear Antibodies (ANA) screen test with a 1.2 ratio, anti-dsDNA IgG positive (25.3 IU/ml), anti-dsDNA IgA positive (26.2 IU/ml) and PM-Scl positive. In contrast, anti-U1 RNP, anti-Mi, anti-Jo-1, anti-SSA, anti-SSB, MDA5, NXP2, and SAE were all negative. Additionally, a comprehensive myositis/antisynthetase panel including PL-7, PL-12, EJ, OJ, was also negative.

#### 2.6.3 Histopathology

Muscle biopsy of the left vastus lateralis demonstrated inflammatory myopathy characterized by patchy endomysial lymphocytic infiltration; without classic perifascicular atrophy.

#### 2.6.4 Imaging studies and PFTs

**FIGURE 2:**
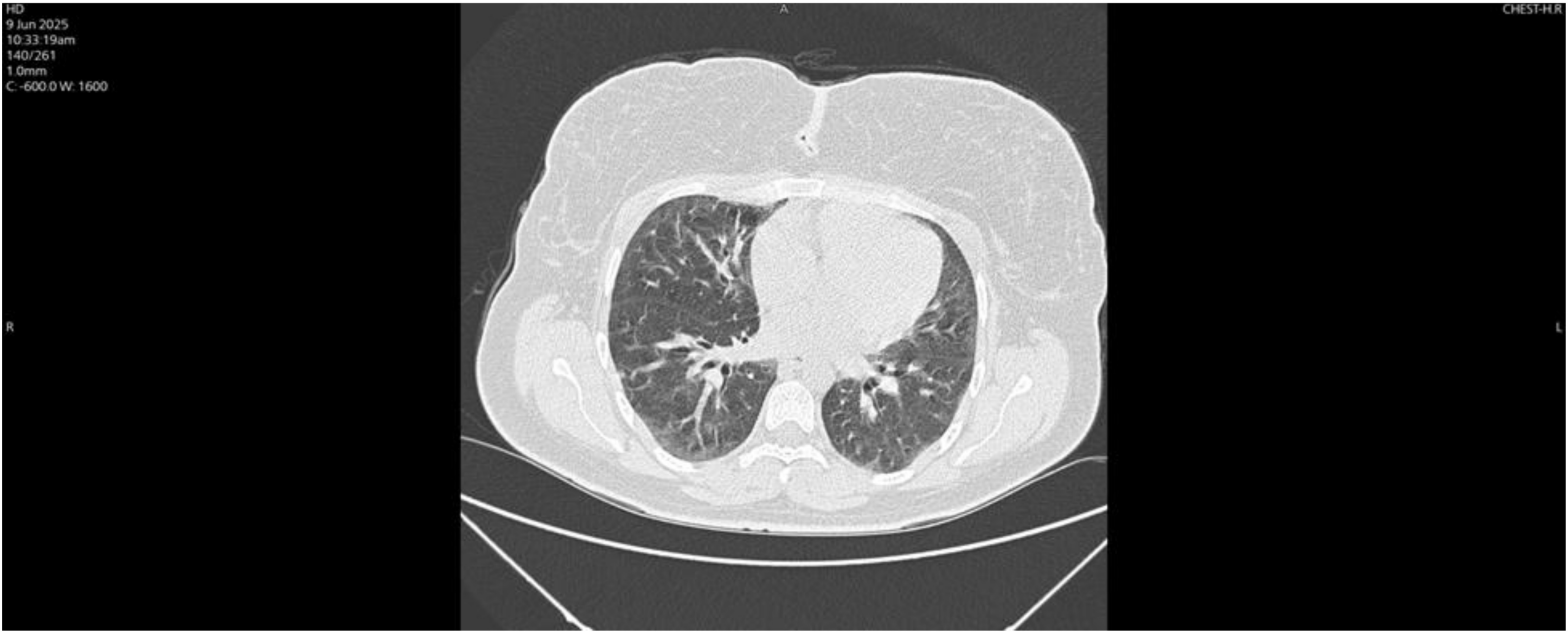
High-resolution chest CT demonstrating bilateral lower-lobe-predominant centrilobular and subpleural ground-glass opacities consistent with inflammatory interstitial lung disease.

Computed tomography (CT) imaging demonstrated hepatomegaly (approximately 19 cm) and splenomegaly (approximately 16 cm) without lymphadenopathy. Additional findings included a 4-cm left ovarian cyst and a BI-RADS 3 left breast lesion on breast ultrasonography, without other significant abnormalities. High resolution chest CT (Figure 2), demonstrated bilateral lower-lobe predominant ground-glass opacities with centrilobular and subpleural interstitial involvement consistent with inflammatory ILD. Pulmonary function tests (PFT) indicated a restrictive pattern: FVC 33.5%, FEV1 37.6%; preserved FVC/FEV1.

The final diagnosis was anti-PM/Scl-positive overlap myositis with dermatomyositis and systemic sclerosis features complicated by inflammatory ILD, based on the combination of classic dermatomyositis cutaneous manifestations, inflammatory myopathy, Raynaud phenomenon, puffy hands, positive anti-PM/Scl antibodies, restrictive interstitial lung disease, and supportive histopathologic findings.

### 2.7 Differential diagnosis

Several inflammatory, autoimmune, metabolic, viral, and paraneoplastic conditions were included in the differential diagnosis for this patient’s presentation. At first, progressive proximal muscle weakness and significantly high creatine kinase values were thought to be signs of polymyositis. Nevertheless, a diagnosis of dermatomyositis rather than polymyositis was supported by the presence of distinctive cutaneous symptoms, such as heliotrope rash and Gottron’s papules (5).

Given the history of PCR-confirmed Influenza B infection during pregnancy, viral myositis, namely influenza-associated myositis, was also taken into consideration. However, due to the multisystem involvement, persistent autoimmune features, chronic progressive course, and absence of spontaneous remission following the acute infection, this diagnosis was deemed improbable (5).

Systemic lupus erythematosus (SLE) was considered due to constitutional symptoms, positive ANA results, and positive anti-dsDNA antibodies (7). Nevertheless, the lack of lupus-specific organ involvement and the dominance of inflammatory myopathy and dermatomyositis-type skin findings suggested against isolated SLE (7).

Systemic sclerosis was contemplated because of Raynaud’s phenomenon, hand swelling, interstitial lung disease, and scleroderma-like skin changes (8). However, the degree of muscle involvement and typical dermatomyositis cutaneous features supported an overlap of connective tissue disease rather than isolated systemic sclerosis (8).

Mixed connective tissue disease (MCTD) was initially considered due to overlapping connective tissue disease features. However, the absence of anti-U1 ribonucleoprotein antibodies argued against classic MCTD (3, 9).

On the other hand, a PM-Scl-associated myositis–systemic sclerosis overlap syndrome with a dermatomyositis phenotype was more likely in the presence of anti-PM-Scl antibodies and clinical features of inflammatory myopathy with systemic sclerosis (10).

Due to proximal muscular weakness, elevated CK, ILD, and mechanic’s-hand–like changes, antisynthetase syndrome was also included in the differential diagnosis (11). However, the presence of classic dermatomyositis skin manifestations (Gottron’s papules and heliotrope rash) and negative extended antisynthetase antibody testing (including anti-PL-7, anti-PL-12, anti-EJ, anti-OJ, and anti-Jo-1) made antisynthetase syndrome less likely (11).

Overall, the clinical and serological profile remained most consistent with anti-PM-Scl-associated overlap myositis rather than antisynthetase syndrome (10).

### 2.8 Treatment

High-dose corticosteroids were introduced as the main treatment approach for severe inflammatory myopathy with systemic involvement (11, 12). Although disease control remained poor, mycophenolate mofetil was later offered as a steroid-sparing drug due to its possible pulmonary advantages in ILD associated with connective tissue disease (12). While CK levels showed a modest decline following treatment initiation, the patient remained clinically symptomatic with persistent dyspnea, and follow-up PFTs demonstrated no significant respiratory improvement. Due to refractory disease activity, overlap characteristics, and substantial functional and respiratory impairment, an escalation to rituximab was necessary, which led to a notable improvement in clinical outcomes. Two 1-g infusions were administered 2 weeks apart After significant symptom relief, methotrexate and folate supplements were eventually started as maintenance therapy.

### 2.9 Outcomes, follow-up, and learning points

**FIGURE 3:**
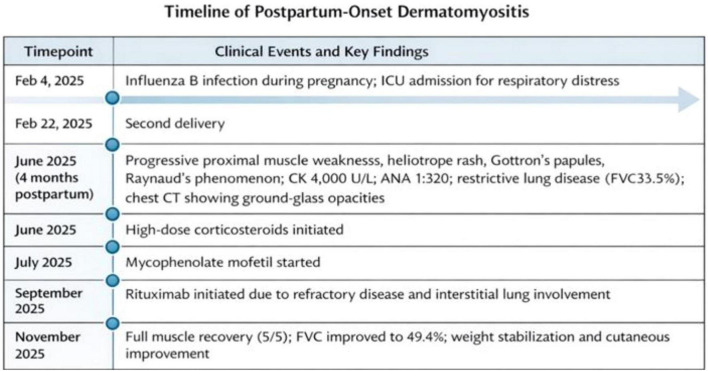
Clinical timeline of postpartum-onset dermatomyositis.

Weight stabilization, complete muscle strength recovery (5/5), significant improvement in cutaneous symptoms, significant improvement in dysphagia, resolution of hepatosplenomegaly on follow-up ultrasound, and an increase in FVC from 33.5% to 49.4% were all indicative of the remarkable clinical response to rituximab therapy. Rituximab therapy was escalated due to prolonged functional, respiratory, and swallowing impairment that persisted after mycophenolate mofetil treatment (12). Testing for pulmonary function throughout mycophenolate therapy revealed a continually poor FVC, although rituximab delivery resulted in a notable improvement. Following an increase in immunosuppressive therapy, creatine kinase levels also gradually improved. The patient continued to take methotrexate and low-dose corticosteroids while maintaining clinical stability (12). Even though the patient’s dysphagia had significantly improved, an upper endoscopy was scheduled, and an esophageal sample showed no signs of granulomatous inflammation, eosinophilic infiltration, dysplasia, or cancer. Ongoing pulmonary monitoring and malignancy surveillance have not yet revealed any signs of progression or occult cancer (11, 12). The clinical response, treatment strategies, diagnostic assessment, and symptom progression over time are summarized in Figure 3. In postpartum presentations of anti-PM/Scl-positive myositis–systemic sclerosis overlap syndrome with a dermatomyositis phenotype, this case emphasizes the significance of rigorous serologic interpretation and recognition of overlap of connective tissue disease manifestations (8, 13).

## 3 Discussion

Dermatomyositis (DM) occurring within CTD overlap syndromes represents a diagnostically complex and heterogeneous clinical entity. In the present case, the patient demonstrated a constellation of clinical manifestations, serologic, and radiologic findings most consistent with an anti-PM/Scl–associated overlap myositis with dermatomyositis and systemic sclerosis overlap features. The coexistence of inflammatory myopathy, restrictive interstitial lung disease, characteristic dermatomyositis cutaneous manifestations, puffy hands, Raynaud phenomenon, and anti-PM/Scl positivity supports classification within the spectrum of PM/Scl-associated overlap syndromes.

Anti-PM/Scl antibodies are well-recognized markers of overlap syndromes combining features of inflammatory myopathy and systemic sclerosis, frequently associated with ILD, Raynaud’s phenomenon, and puffy hands (13, 14). While polymyositis is more commonly described in this context, dermatomyositis phenotypes have increasingly been reported, often with atypical or systemic manifestations (14, 15). In this patient, the presence of classic dermatomyositis cutaneous findings, including heliotrope rash and Gottron’s papules, together with markedly elevated creatine kinase levels and biopsy-confirmed inflammatory myopathy without perifascicular atrophy, highlights the variability of histopathologic findings in overlap syndromes (16). This reinforces the importance of integrating clinical, serologic, and histopathologic data when establishing a diagnosis.

An important differential diagnosis in this case was antisynthetase syndrome, given the coexistence of ILD, proximal muscle weakness, and hyperkeratotic fissuring of the hands suggestive of mechanic’s hands (17). However, a comprehensive antisynthetase antibody panel, including anti-Jo-1, PL-7, PL-12, EJ, and OJ, was negative, making this diagnosis less likely (18). The availability and performance of extended serologic testing in this patient strengthen diagnostic confidence and address a common limitation in similar reports where incomplete antibody panels may lead to diagnostic uncertainty (19).

An additional unusual feature in this case was the existence of anti-dsDNA positive in the absence of clinical lupus symptoms (20). Although anti-dsDNA antibodies are typically associated with systemic lupus erythematosus, isolated positivity has been observed in overlap of connective tissue disorders and inflammatory myopathies. Despite positive serology, the patient’s lack of lupus nephritis, cytopenias, serositis, hypocomplementemia, or other SLE-defining organ involvement ruled out isolated SLE. This finding could represent broader immunological dysregulation within overlap disorders rather than a discrete lupus manifestation (21).

The temporal association between disease onset and the postpartum period suggests a potential immunological trigger. Pregnancy is characterized by a predominantly T-helper 2 (Th2)-mediated immune state that promotes maternal tolerance toward the fetus (22). Following delivery, this state shifts toward a T-helper 1 (Th1)-dominant pro-inflammatory profile, a process referred to as postpartum immune reconstitution (23). This transition has been implicated in the onset or exacerbation of autoimmune diseases, including inflammatory myopathies (24). In this case, symptom onset approximately four months postpartum supports this mechanism. Additionally, the patient’s preceding influenza B infection during pregnancy may have served as a secondary immunologic trigger; however, the chronic and progressive nature of her symptoms, along with persistent autoimmune features, argues against a purely post-viral myositis (25).

Pulmonary involvement represents one of the most clinically significant aspects of CTD overlap syndromes and is a major determinant of morbidity and mortality (26). The patient exhibited restrictive lung disease with markedly reduced FVC and high-resolution CT findings of bilateral ground-glass opacities. Importantly, follow-up pulmonary function testing demonstrated improvement in FVC from 33.5% to 49.4% after escalation to rituximab therapy, supporting a predominantly inflammatory rather than fibrotic ILD phenotype (27). This observation is consistent with early CTD-associated ILD, where inflammatory alveolitis may be reversible with timely immunosuppressive therapy, in contrast to established fibrotic disease, which is typically irreversible.

The presence of hepatosplenomegaly and significant unintentional weight loss introduced additional diagnostic complexity and raised concern for malignancy-associated myositis and macrophage activation syndrome (MAS) (28). However, the absence of cytopenias, hyperferritinemia, or other laboratory markers suggestive of MAS, along with a negative hematologic evaluation, makes this diagnosis unlikely (29). Similarly, malignancy workup revealed only a BI-RADS 3 breast lesion without evidence of systemic malignancy. In this context, hepatosplenomegaly is more plausibly explained as a reactive manifestation of systemic inflammation. Notably, anti-PM/Scl–associated myositis has been reported to carry a relatively lower malignancy risk compared to other myositis-specific antibodies; however, continued surveillance remains essential given the established association between dermatomyositis and malignancy (28, 30).

From a therapeutic standpoint, this case highlights the challenges of managing overlap myositis with multisystem involvement. While systemic corticosteroids remain the cornerstone of initial therapy, response is often incomplete in overlap syndromes (31). In this patient, treatment with mycophenolate mofetil resulted in only partial clinical improvement, with persistent muscle weakness, pulmonary dysfunction, and dysphagia. Notably, clinical, functional, and pulmonary outcomes improved significantly following escalation to rituximab, including resolution of dysphagia and improvement in muscle strength and pulmonary function. This clinical course suggests that disease control was suboptimal on mycophenolate therapy and improved following B-cell–targeted treatment, rather than reflecting disease worsening on prior therapy.

The favorable clinical response to rituximab may support a role for B-cell–mediated immune mechanisms in overlap syndromes (32). Postpartum immune reconstitution is also associated with increased B-cell activation and expansion of autoreactive clones, providing a plausible immunologic explanation for both disease onset and therapeutic response (33). These findings are consistent with emerging evidence supporting rituximab as an effective treatment option in refractory inflammatory myopathies and CTD-associated ILD (34–36).

To contextualize this case, previously reported studies and case reports of postpartum-onset dermatomyositis and overlap syndromes were reviewed (Supplementary Table 1). These reports demonstrate considerable heterogeneity in clinical presentation, serologic profiles, and outcomes (37). For example, cases of postpartum dermatomyositis have been described with anti-MDA5 and anti-Jo-1 antibodies, frequently associated with ILD and systemic manifestations, and generally responsive to immunosuppressive therapy (38, 39). It should be noted that the literature comparison presented in (Supplementary Table 1) was derived from a non-systematic review of available published cases and should not be interpreted as an exhaustive representation of the literature.

Anti-PM/Scl–positive postpartum overlap syndromes are less frequently described in the available literature, and the combination of dermatomyositis phenotype, ILD, and postpartum onset has been reported in only a limited number of cases. Rather than emphasizing the rarity of this presentation, this case contributes several clinically relevant teaching points: the importance of postpartum immune reconstitution as a trigger for autoimmune disease, the potential reversibility of inflammatory ILD with timely immunosuppression, and the role of rituximab in refractory overlap myositis with pulmonary involvement. Additionally, observational data suggest that rituximab may be beneficial in CTD-associated ILD, further supporting its use in refractory cases (34, 35).

Importantly, in the absence of a formal systematic search methodology, claims regarding the rarity or uniqueness of this presentation should be interpreted cautiously. Rather than emphasizing novelty alone, this case highlights several important clinical considerations: (1) the importance of precise serologic classification in characterizing overlap CTD phenotypes, (2) the recognition of postpartum immune shifts as potential triggers of autoimmune disease, (3) the potential reversibility of inflammatory ILD with early and appropriate immunosuppression, and (4) the role of rituximab in managing refractory overlap myositis with pulmonary involvement.

## 4 Conclusion

This case illustrates the diagnostic complexity of postpartum-onset anti-PM/Scl–associated dermatomyositis–systemic sclerosis overlap syndrome with multisystem involvement including inflammatory interstitial lung disease (ILD), in a young patient (13). It underscores the diagnostic challenges inherent to overlap connective tissue diseases, particularly when clinical manifestations span multiple connective tissue disease spectra reflecting the diagnostic complexity of overlap CTDs (40).

This report emphasizes the importance of comprehensive serologic evaluation, including extended myositis and antisynthetase antibody panels, in accurately characterizing overlap syndromes and avoiding diagnostic ambiguity (19). The temporal association with the postpartum period further supports the role of immune reconstitution as a potential trigger for autoimmune disease onset, an important consideration in similar clinical scenarios (22). Importantly, this case demonstrates that early inflammatory ILD in the context of overlap syndromes may be reversible with prompt escalation of immunosuppressive therapy (30). The marked clinical, functional, and pulmonary improvement observed following rituximab therapy highlights the potential role of B-cell–targeted treatment in refractory overlap myositis, particularly in patients with persistent systemic and pulmonary involvement despite conventional immunosuppression (34, 35).

Rather than focusing solely on rarity, this case contributes important clinical observations into the recognition, classification, and management of overlap connective tissue diseases, and supports an individualized therapeutic approach in complex presentations.

## 5 Limitations

This report has several limitations that should be acknowledged. First, as a single case report, the findings are inherently limited in generalizability and cannot establish causal relationships or treatment efficacy. Second, although an extensive serologic evaluation was performed, including a comprehensive myositis and antisynthetase panel, autoantibody profiles in connective tissue diseases may evolve over time; requiring continued longitudinal follow-up.

Third, while the manuscript includes a literature comparison (Supplementary Table 1), a formal systematic search strategy was not performed, which limits the ability to make definitive claims regarding the rarity or uniqueness of this presentation. Consequently, the findings should be interpreted in the context of available reported cases rather than as an exhaustive representation of the literature.

Additionally, certain aspects of the clinical course, such as the contribution of prior therapies (e.g., mycophenolate mofetil) versus rituximab to overall improvement, cannot be fully disentangled, although the temporal association suggests a more pronounced response following B-cell–targeted therapy (35). Similarly, while hepatosplenomegaly was interpreted as a reactive inflammatory manifestation, the absence of histopathologic confirmation limits definitive conclusions regarding its etiology.

Finally, long-term follow-up data remain limited, particularly with respect to disease progression, relapse risk, and malignancy surveillance, which are important considerations in patients with dermatomyositis and overlap syndromes (41).

## Data Availability

The original contributions presented in this study are included in the article/Supplementary material, further inquiries can be directed to the corresponding author.
