# Correction: Postpartum-onset anti-PM/Scl–positive dermatomyositis–systemic sclerosis overlap syndrome with reversible interstitial lung disease: a case report

**DOI:** 10.3389/fmed.2026.1928113

**Published:** 2026-07-16

**Authors:** Joud Zghyer, Asad Omarion, Leen Zghyer, Yaman Ayasa, Omar Al-Deek, Adnan A. M. Wahdan

**Affiliations:** 1 Faculty of Medicine, Al-Quds University, Jerusalem, Palestine; 2 Department of Internal Medicine, Palestine Medical Complex, Ramallah, Palestine

**Keywords:** dermatomyositis, interstitial lung disease, overlap syndrome, postpartum, rituximab, systemic sclerosis

Affiliation Faculty of Medicine, Al-Quds University, Jerusalem, Palestine was erroneously omitted for author Adnan A. M. Wahdan. This affiliation has now been added, and the corresponding author's affiliations should read: Faculty of Medicine, Al-Quds University, Jerusalem, Palestine; Department of Internal Medicine, Palestine Medical Complex, Ramallah, Palestine.

The original version of this article has been updated.

