# Supplementary material for: Postpartum-onset anti-PM/Scl–positive dermatomyositis–systemic sclerosis overlap syndrome with reversible interstitial lung disease: a case report
Source: Front Med (Lausanne). 2026 Jul 1;13:1848783. doi: 10.3389/fmed.2026.1848783 (PMC13368723; doi:10.3389/fmed.2026.1848783)
Supplement: Supplementary file 1 [file Table_1.DOCX]

**Table S1.** Literature review on Dermatomyositis, overlap syndromes, studies with emphasis on postpartum onset and serology.

| **Author (year)** | **Study type** | | **Patient Characteristics** | **Postpartum onset** | **CTD overlap** | **ILD** | **Autoantibodies** | **Systemic Manifestation** | **Treatment and Outcomes** |
| --- | --- | --- | --- | --- | --- | --- | --- | --- | --- |
| Troyanov et al. (2014) [15] | Cohort study | Dermatomyositis and overlap myositis patients | | No Presentation | Yes | Present in overlap group | Myositis antibodies (incl. DM-related) | Muscle + systemic CTD features (e.g., arthritis, Raynaud’s) |  |
| Kawamata et al. (2026) [38] | Case report | 19-year-old male with dermatomyositis phenotype | | No Presentation | Possible | Rapidly progressive ILD | Anti-PM/Scl positive | Severe systemic disease dominated by ILD + myositis |  |
| Saito et al., 2019 [42] | Case Report | | 27-year-old, with inflammatory myopathy | During pregnancy | No | Yes | Anti-Jo-1 Positive | Dyspnea, Proximal muscle weakness, and arthritis | Prednisolone and cyclosporine- Improved |
| Chiang et al., 2021 [39] | Cohort | | Patients with Inflammatory myopathy | No presentation | Yes | Present in some | Variable anti -U1-RNP | Variable including ILD, Raynaud phenomenon and proximal muscle weakness | Immunosuppressive medications- Variable outcome |
| Seedat et al., 2024 [40] | Observational | | CTD-ILD patients | No presentation | Yes | Severe ILD | No Information provided | Variable but mainly including ILD, dyspnea | Rituximab- Patients improved |
| Wanzenried et al., 2022 [41] | Review | | MCTD patients | Rare presentation | Yes | Yes | Positive anti-U1-RNP | Variable | Immunosuppressive therapy with variable outcome |
| Present Case | Case Report | | 25-year-old woman with positive PM/Scl Antibody- Dermatomyositis and overlap syndrome | 4 months | Yes | Yes | Positive ANA, anti-dsDNA, Anti PM-Scl, Negative Anti-U1-RNP antibody | ILD, Hepatosplenomegaly, unintentional weight loss, Proximal muscle weakness | High-dose corticosteroids, Mycophenolate mofetil and Rituximab- The patient improved |
