# Supplementary material for: Postpartum-onset anti-PM/Scl–positive dermatomyositis–systemic sclerosis overlap syndrome with reversible interstitial lung disease: a case report
Source: Front Med (Lausanne). 2026 Jul 1;13:1848783. doi: 10.3389/fmed.2026.1848783 (PMC13368723; doi:10.3389/fmed.2026.1848783)
Supplement: Supplementary file 2 [file Table_2.DOCX]

**Table S2.** Literature review on Dermatomyositis, MCTD overlap syndromes, studies with emphasis on postpartum onset and serology.

| **Author (year)** | **Study type** | **Patient Characteristics** | **Postpartum onset** | **CTD overlap** | **ILD** | **Autoantibodies** | **Systemic Manifestation** | **Treatment and Outcomes** |
| --- | --- | --- | --- | --- | --- | --- | --- | --- |
| Shimizu et al., 2021  [34] | Case Report | 33-year-old, with amyopathic dermatomyositis | 3 months | No | Yes, rapidly progressive | Anti-MDA5 positive | Dyspnea, hypoxemia and skin rash | High- dose steroids, tacrolimus and cyclophosphamide- The patient clinically improved |
| Saito et al.,2019 [35] | Case Report | 27-year-old, with inflammatory myopathy | During pregnancy | No | Yes | Anti-Jo-1 Positive | Dyspnea, Proximal muscle weakness, and arthritis | Prednisolone and cyclosporine- Improved |
| Chiang et al., 2021 [17] | Cohort | Patients with Inflammatory myopathy | No presentation | Yes | Present in some | Variable anti -U1-RNP | Variable including ILD, Raynaud phenomenon and proximal muscle weakness | Immunosuppressive medications- Variable outcome |
| Seedat et al., 2024 [31] | Observational | CTD-ILD patients | No presentation | Yes | Severe ILD | No Information provided | Variable but mainly including ILD, dyspnea | Rituximab- Patients improved |
| Wanzeried et al., 2022 [2] | Review | MCTD patients | Rare presentation | Yes | Yes | Positive anti-U1-RNP | Variable | Immunosuppressive therapy with variable outcome |
| Present Case | Case Report | 25-year-old woman with Dermatomyositis and overlap syndrome | 4 months | Yes | Yes | Positive ANA, anti-dsDNA, Anti PM-Scl, Negative Anti-U1-RNP antibody | ILD, Hepatosplenomegaly, unintentional weight loss, Proximal muscle weakness | High-dose corticosteroids, Mycophenolate mofetil and Rituximab- The patient improved |
